# Reconstructing the hydraulics of the world’s first industrial complex, the second century CE Barbegal watermills, France

**DOI:** 10.1038/s41598-020-74900-5

**Published:** 2020-10-21

**Authors:** Cees W. Passchier, Marcel Bourgeois, Pierre-Louis Viollet, Gül Sürmelihindi, Vincent Bernard, Philippe Leveau, Christoph Spötl

**Affiliations:** 1 Department of Earth Sciences, Johannes Gutenberg University, 55128 Mainz, Germany; 2 Vicat Group, 38081 L’Isle d’Abeau Cedex, France; 3 Société Hydrotechnique de France, 25 rue des Favorites, 75015 Paris, France; 4 CNRS, UMR 6566 CReAAH, Université Rennes 1, Rennes, France; 5 CNRS, CCJ, UMR 7299, Aix Marseille Université, 13094 Aix-en-Provence, France; 6 Institute of Geology, University of Innsbruck, Innrain 52, 6020 Innsbruck, Austria

**Keywords:** Hydrology, Energy infrastructure, Sedimentology, Applied physics, Techniques and instrumentation

## Abstract

The Barbegal watermill complex, a unique cluster of 16 waterwheels in southern France, was the first known attempt in Europe to set up an industrial-scale complex of machines during the culmination of Roman Civilization in the second century CE. Little is known about the state of technological advance in this period, especially in hydraulics and the contemporary diffusion of knowledge. Since the upper part of the Barbegal mill complex has been destroyed and no traces of the wooden machinery survived, the mode of operation of these mills has long remained elusive. Carbonate incrustations that formed on the woodwork of the mills were used to reconstruct its structure and function, revealing a sophisticated hydraulic setup unique in the history of water mills. The lower mills used an elbow shaped flume to bring water onto overshot millwheels. This flume was specially adapted to the small water basins and serial arrangement of the mills on the slope. Carbonate deposits from ancient water systems are therefore a powerful tool in archaeological reconstructions and provide tantalizing insights into the skills of Roman engineers during a period of history that is the direct predecessor of our modern civilization.

## Introduction

**Figure 1:**
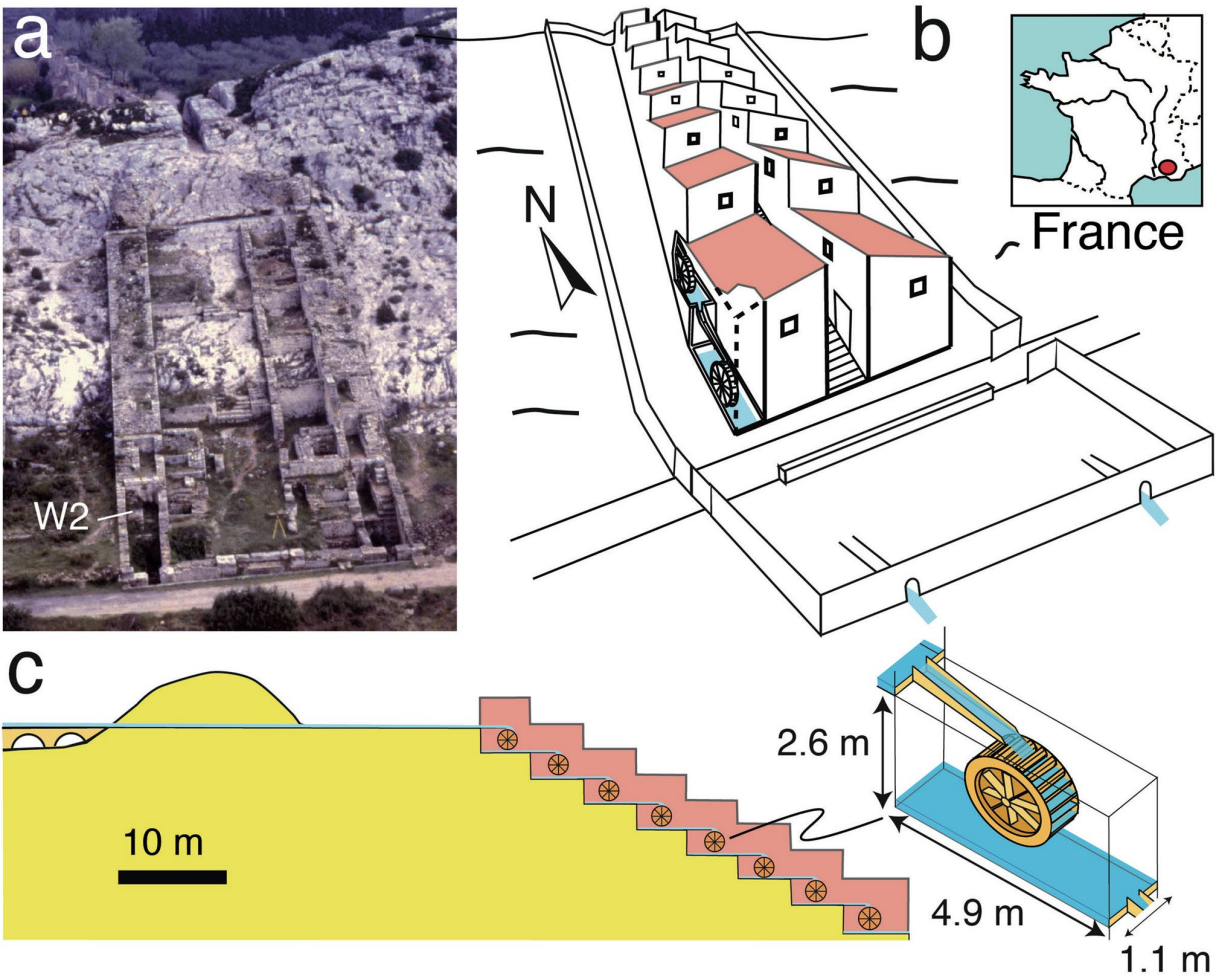
The Barbegal watermill complex, built in the second century CE in Southern France. (**a**) Ruins of the complex photographed in 1996, seen from the south. The aqueduct feeding the mills entered from the north through a rock cut seen at the top. W2 is the mill basin where carbonate was found in the axle window. The elbow flume was probably located in this basin, or the one below. (**b**) Reconstruction of the two parallel trains of eight mills (of which two are shown) that were built on a natural slope, with central buildings. Inset shows the location in France. (**c**) N–S cross-section of the mill complex along one train of millwheels, drawn to scale, with a close-up drawing of one mill.

Roman Imperial society is recognized to have been an economic powerhouse similar to our modern, globalized society in many ways^1^. Strong economic growth accompanied construction of an impressive infrastructure including roads, harbors, cities and mines with associated environmental impact and significant pollution, e.g. of lead, recorded even in remote regions such as central Greenland^2^. Less well known is that Roman engineers specifically excelled in the construction of hydraulic infrastructure such as water supply systems, norias and watermills with a technical standard that was only reached again in the sixteenth century^3–6^. From as early as the first century BC, watermills were among the first energy sources not depending upon human or animal muscle power^7,8^. In Roman civilization, they were crucial for increasing the production of flour, to cut wood and stone, and to process ore^1,9–11^. The Roman watermill complex of Barbegal in Southern France from the second century CE is an outstanding example of this development as one of the first industrial complexes in European history, displaying the greatest known concentration of mechanical power in the ancient world^12,13^. It is a unique arrangement of 16 waterwheels in two parallel rows of eight mills, separated by central buildings and fed by an aqueduct (Fig. 1b,c)^12–14^. Since the upper parts of the mill complex have been destroyed and no traces of the wooden machinery and its water supply system survived, the type of millwheels, and its operation mode have long remained elusive.

Fortunately, during mill operation, calcium carbonate precipitated on those parts of the wooden mill machinery that were in contact with water. Freshwater carbonate deposits in ancient water systems have been used to reconstruct environmental conditions^15,16^ and to recognize local extreme events such as floods and earthquakes^17–19^. Moreover, they are suited to obtain archaeological information on topics such as water system usage^15,20,21^, identification of springs used^22^, number of years of aqueduct operation^15,20,23^, aqueduct cleaning and maintenance^24–26^, restructuring of water systems^27,28^ and a decrease in maintenance and final abandonment^24,29–31^.

In Barbegal, carbonate deposits have survived as casts of the original timber, which can provide detailed information about individual mill parts, shedding light on the operation of the complex^29,30^. For most carbonate fragments, it was possible to reconstruct on which mill parts they formed, but the origin of some pieces remained ambiguous. This paper deals with some enigmatic carbonate fragments that formed inside an elbow-shaped mill flume that may be of crucial importance to understand the function of the entire complex. Detailed analysis and reconstruction of its most likely position and function revealed that this flume has no equivalent in ancient, medieval or modern mills.

## Results

### The elbow-flume

**Figure 2:**
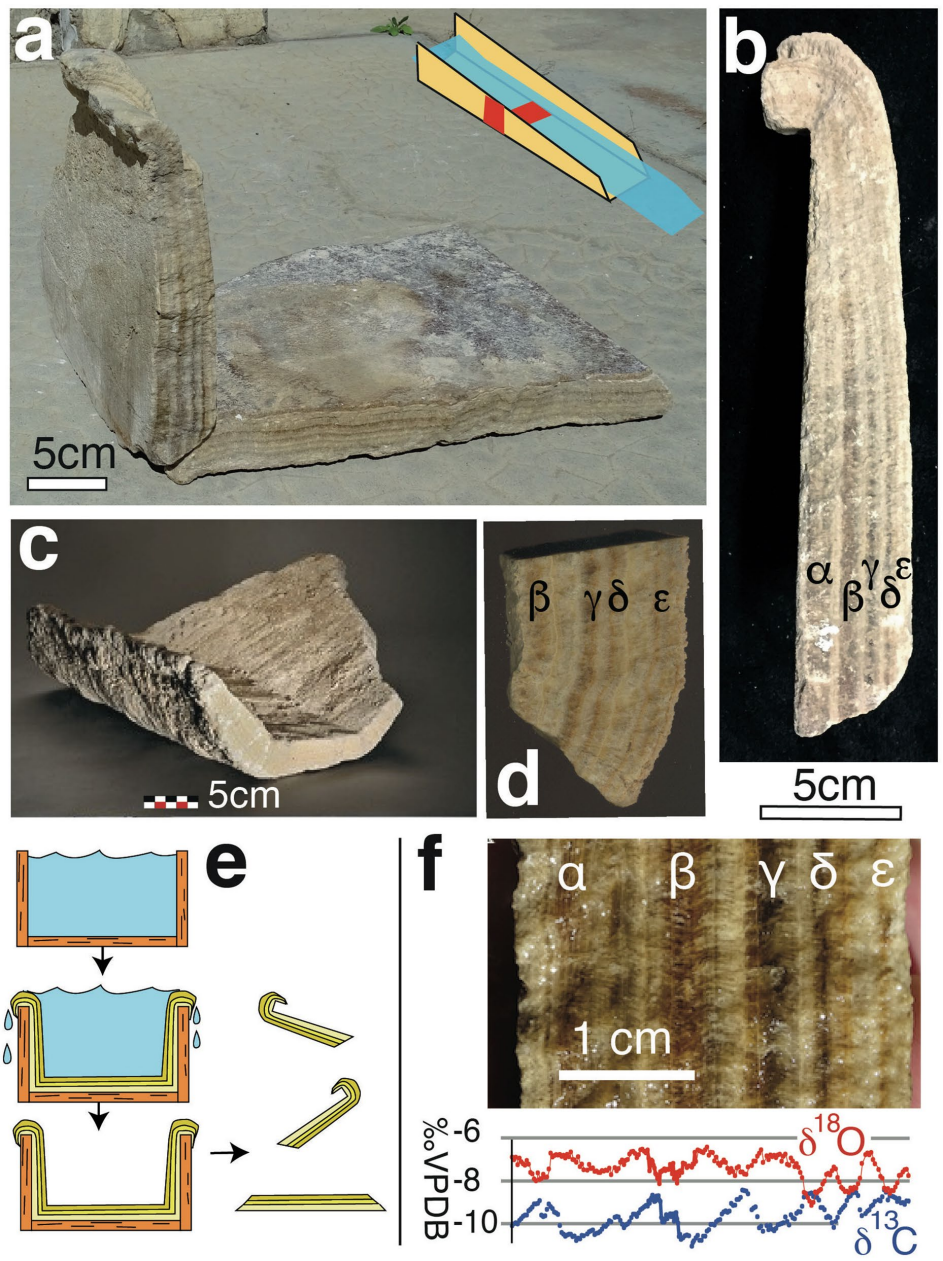
Carbonate deposits formed during operation of the Barbegal watermills. Individual fragments are labelled N(-). The sides or cross-sections of these fragments, where the internal stratigraphy is visible, are labelled f(-). (**a**) Carbonate deposits originating from a wooden flume. A bottom and a sidewall fragment are shown in their original arrangement. Inset shows the position of the fragments in a flume. (**b**) Side f2 of carbonate wall fragment N55 with an overhang deposit at the top. a–ε indicate individual layers. (**c**) Carbonate fragment N138, presumably derived from the bucket of a mill wheel. (**d**) Stratigraphy of fragment N138. (**e**) Reconstruction of carbonate deposition in an overflowing flume. When the channel had been dismantled or the wood had decomposed, deposits broke into bottom and sidewall segments. (**f**) Stratigraphy of a flume in fragment N49 and corresponding stable isotope profile of carbon (δ^13^C) and oxygen (δ^18^O). The antithetic cycles of δ^13^C and δ^18^O represent seven years of deposition and flume operation, starting and ending in winter.

Most of the surviving large fragments of carbonate deposits from the Barbegal mills formed inside wooden millrun flumes, the elevated gutters that once transported water to the mill wheels (Fig. 2a,b)^29,30^. Some carbonate fragments could be attributed to the waterwheels themselves (Fig. 2c). When the mills were abandoned and the wood had decomposed, the carbonate crusts that had formed inside flumes broke into trapezoid- and hook-shaped fragments that once covered the bottom and side walls, respectively (Fig. 2a,b,e). Many hook-shaped fragments have a curved “overhang” above the impression of the woodwork on the carbonate crust (Fig. 2a,b), indicating that the flumes had been completely filled and water was overflowing their side boards.

**Figure 3:**
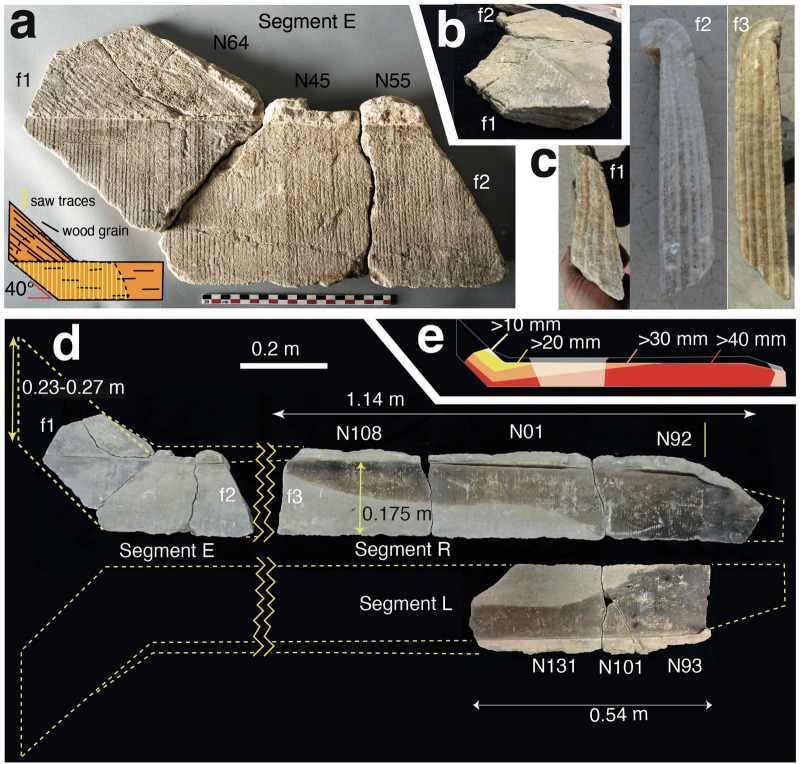
Carbonate segments of the Barbegal elbow flume. Segments are composed of individual fragments, labelled N(-). The sides or cross-sections of these fragments, where the internal stratigraphy is visible, are labelled f(-). (**a**) Carbonate segment E. This crust originally covered two adjacent planks, showing two different directions of wood grain and sawing patterns, as shown in the inset. (**b**) Segment E seen from the top left side, showing thinning of the carbonate deposit towards the top. (**c**) Cross-sections of the sides f1 and f2 of segment E and side f3 of segment R expose the stratigraphy. (**d**) Reconstruction of the elbow-shaped arrangement of segments E, R and L. (**e**) Color-coded relative thickness of carbonate along segments E and R. Yellow to red indicates increasing thickness. White sections are missing.

One carbonate segment composed of three fragments (Segment E) drew our attention because of its unusual elbow-shape (Fig. 3a). It formed on two planks, fitted together at an angle of 40° ± 1° on a right-hand flume sidewall, with overflow deposits. The impression on the longer side of the segment shows fine, 4.8–5.2 mm wide regularly spaced sawing traces normal to the grain of the wood. The regular spacing and straight nature of these traces suggests that they were produced by mechanical sawing^30^. To our knowledge, this presents the oldest known record of the use of a mechanical saw to cut wood in history.

Segment R, another flume sidewall cast, has identical overflow deposits and sawing traces as segment E (Fig. 3d). In cross-section along their sides, fragments from E and R also show the same stratigraphy and asymmetric geometry (cross sections f2 and f3 in Fig. 3c; Supplementary Fig. S1). Segment R was mostly deposited against a single plank, with the impression of a second plank at one extremity showing a different sawing pattern (Fig. 3d, fragment N92). At the top of this second plank, the overflow deposits of the gutter start bending down, indicating a decreasing height of the sidewall towards the right. This is typical of the nozzles of flumes where they feed water onto a waterwheel.

Segment L, composed of three fragments (Fig. 3d), has the same sawing traces, carbonate stratigraphy and plank height (0.175 ± 0.003 m) as segments R and E. L and R show mirror symmetry: on one side L has a similar reduction in height of the side wall as segment R, indicating the onset of a nozzle. Segments R and L are partly covered with a black veneer that postdates carbonate deposition, since it is also present in some of the fractures (Fig. 3d, Supplementary Fig. S1). This veneer is most likely the result of partial burial of the segments in soil for some years after abandonment of the mill. The continuation of the veneer border along individual fragments (N108/N01/N92 and N131/N101/N93) and the matching patterns of segments R and L suggest that they were still adjacent while they were partially buried (Fig. 3d; Supplementary Fig. S1).

Since segments E, R and L display identical carbonate stratigraphy, plank height, sawing traces and veneer patterns, they are thought to have been deposited on the left- and righthand side of a structure referred to as the “elbow-flume”. All three segments show an identical stratigraphy of alternating brown and white layers, marked (α)–(ε) (Fig. 2b,f). This stratigraphy is identical to that observed in the axle window of mill wheel pit W2 (Fig. 1a)^30^, suggesting that the elbow-flume was employed on the west side of the mill complex: deposits in the eastern wheel pits of the mill complex show different stratigraphies^30^. A fragment, presumably from a bucket of a millwheel with a stratigraphy similar to the elbow-flume was probably derived from the same western mill train (Fig. 2c,d). The bucket was installed after the elbow-flume had already been in operation for some time, since the first layer α is missing (compare Fig. 2d and f).

Figure 2f shows stable isotope profiles of oxygen and carbon measured across the flume stratigraphy. Cyclical changes in δ^18^O are thought to reflect seasonal temperature fluctuations of the aqueduct water^15,16^ with high values (around -6 ‰) characterizing calcite formed during winter. Anti-correlated cyclicity of δ^13^C is attributed to seasonal changes in the CO_2_ degassing rate or biological activity^15,16^. The δ^18^O and δ^13^C patterns indicate that the carbonate deposits on the elbow-flume formed over a period of seven years, starting and ending in winter.

Although the isotope diagram (Fig. 2f) shows that the stratigraphy of the flume is uninterrupted^29^, the stratigraphic thickness along the flume is variable (Fig. 2b). A maximum thickness of 48 mm is reached at the bottom of the flume (Fig. 3e; Supplementary Fig. S1). Near the end of the flume, thickness decreases only slightly upwards to the overhanging top, but at the start of the flume in cross section f1, it decreases markedly, down to 10 mm in the broken top of segment E (f1 in Fig. 3d,e). The overhanging overflow carbonate similarly decreases in thickness from the end of the flume to segment E and is absent from the thin top of the short leg at f1 (Fig. 3). The uninterrupted nature of the layering implies that these thickness gradients formed during carbonate deposition. On the scale of individual layers, β and δ remain nearly constant in thickness and continue into the overhang deposit in the downstream part of the flume (Figs. 2b, 3c), suggesting that water was overflowing the flume sidewalls there during operation (Fig. 2e). The first layer α, however, tapers to the top, and is not present in the overhang deposit (Figs. 2b, 3c). This indicates that after the first year of deposition, water in the elbow-flume was regularly overflowing the sideboards along most of its length, except for the short leg (N64) of segment E, where water depths were persistently lower (Fig. 3a,e).

### Operation of the mills

On each side of the Barbegal mills, mill wheels operated in a train of eight, using water from each upstream wheel pit basin to feed the millwheel downstream (Fig. 1c). Using information on the shape of the elbow-flume, the water depth attained in it, and the known dimensions of the wheel pits, the geometry and arrangement of the mill structures could be reconstructed.

**Figure 4:**
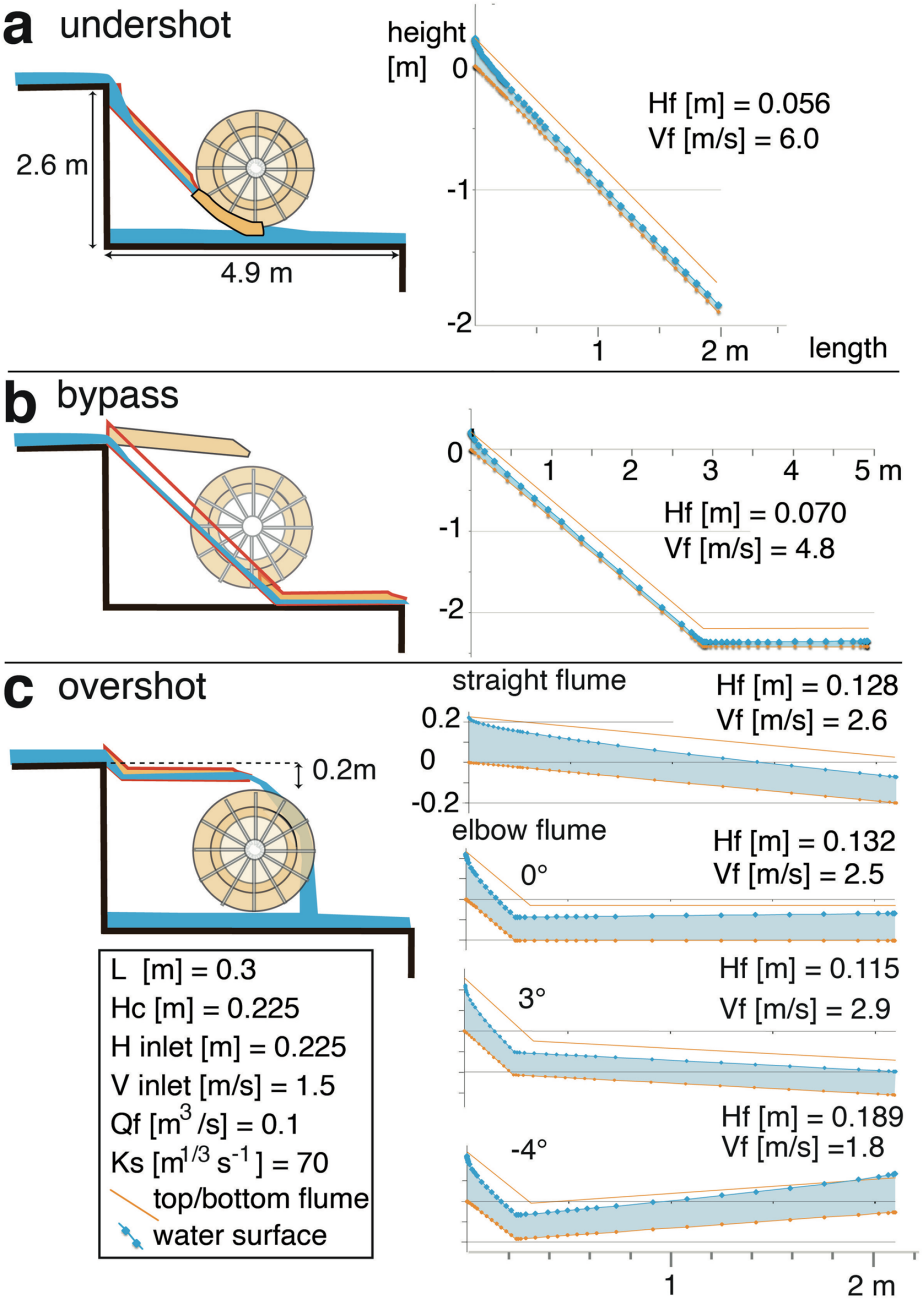
Three plausible models to place an elbow-flume in the wheel pits of the Barbegal complex with matching hydraulic models at right. Outflow water depth (*H*_*f*_) and outflow flow speed (*V*_*f*_) were calculated based on the geometries shown and the parameters given in the inset such as internal width of the flume (L), discharge (flow rate) in the flume *Q*_*f*_ and roughness coefficient *Ks*, considering that the flow at the entrance of the flume is critical (*H*_*inlet*_ = *H*_*c*_). Flow becomes supercritical in the flume. (**a**) Reconstruction of the elbow-flume in an undershot wheel model. (**b**) Reconstruction of the elbow-flume as a bypass that serves as outlet in an overshot model; (**c**) Overshot wheel models. Four hydraulic models are shown: for an inclined straight flume; a horizontal elbow-flume ending at the same level of − 0.2 m; and two elbow flumes with a slope of 3° and a counter slope of − 4°. These models show the effects of variation in shape and dip of a 2.1 m long flume for an overshot mill. Length indication refers to length along the bottom of the entire flume. For the top of the elbow flumes, the shape of segments E and R was used. Flow is supercritical in all cases, and the water height is lower than the critical depth *H*_*c*_. For further explanation see text.

**Figure 5:**
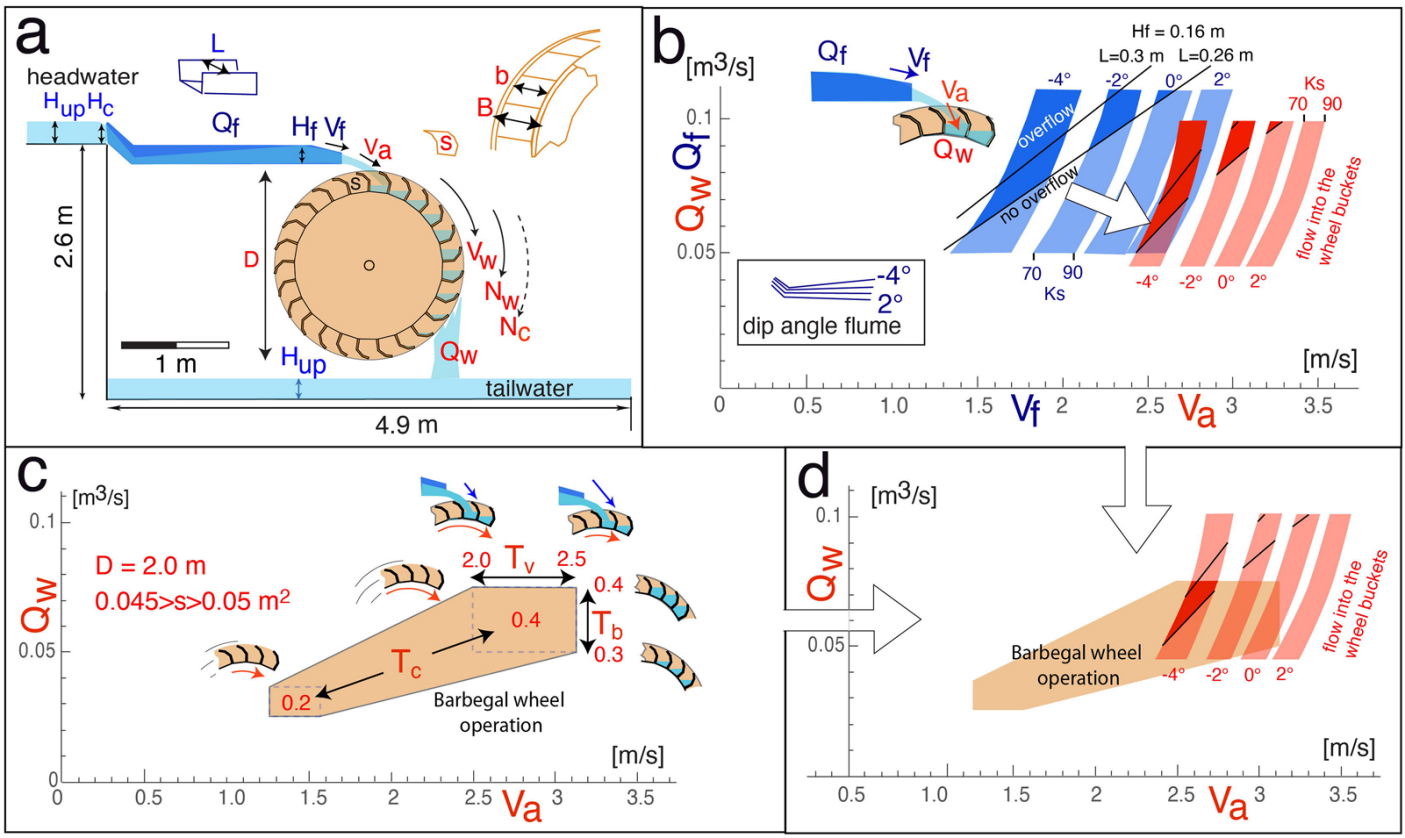
Millwheel, basin and flume geometry with various parameters used to calculate the functioning of overshot mill wheels (parameters explained in Table 1). Parameters pertinent to the flume given in blue, those pertinent to the mill wheel in red. (**a**) General setting and parameters. (**b**) Flume outlet velocity *V*_*f*_ was calculated as a function of flume discharge *Q*_*f*_ for different slopes (− 4° to + 2°) of the elbow-flume runout leg, and an error range in flume roughness factor *Ks*, shown as blue bands. Two black lines give the theoretical relationship between *Q*_*f*_ and *V*_*f*_ at which an estimated overflow depth of *H*_*f*_ = 0.16 m is reached for a clean flume (*L* = 0.3 m) and one partly blocked by carbonate deposition (*L* = 0.26 m); dark blue sections represent overflow conditions. The velocity of water falling 0.2 m onto the wheel *V*_*a*_ was derived from *V*_*f*_ and the angle of the outrun leg of the flume and plotted as red bands. *Q*_*w*_ is assumed to be 10% lower than *Q*_*f*_ due to spillage. Domains of H_f_ > 0.16 are indicated by dark red sections. (**c**) brown polygon presents the range of *V*_*a*_ for discharge *Q*_*w*_ transported by the wheel for optimal operating conditions of a mill wheel served by the elbow flume. These conditions are defined by a range of parameters *s, T*_*c*_*, T*_*b*_ and *T*_*v*_ specifically for the Barbegal mills. *s* is the surface area of the buckets in cross section; *T*_*c*_ describes the ratio of *N*_*w*_ to the critical upper wheel velocity *N*_*c*_; *T*_*v*_ the ratio of the velocity of water striking the wheel *V*_*a*_ to wheel velocity *V*_*w*_; and *T*_*b*_ the filling ratio of wheel buckets. (**d**) Combination of (**b**) and (**c**) shows that *V*_*a*_ and *Q*_*w*_ calculated for the elbow flume agree with values calculated for the Barbegal wheel over a small range in the dark red overlapping field.

Observations and interpretations were fitted to different reconstruction models (Fig. 4). For the estimated wheel diameter (methods; Table1), the watermills of Barbegal could have been either undershot (Fig. 4a) or overshot (Fig. 4c) depending on the way water was fed to the mill wheel and on discharge (flow rate)^32^. Modern overshot wheels are considered to be more energy-efficient than undershot mills^32–34^. Although some authors suggested an undershot geometry for the Barbegal mills^35,36^, the mills have usually been interpreted as overshot, based on the steepness of the slope of the hill on which they were built and the shape of the preserved mill wheel pits^12,13,37–41^. An available discharge of less than 0.13 m^3^/s for each mill train^42^ and the available head of 2.4–2.6 m also favours an overshot geometry^32,33^. Alternatively, the elbow-flume could have served as a bypass along the bottom of a wheel pit basin where either no mill wheel was present, or a wheel was not in use (Fig. 4b). For all models, the possible arrangement of the elbow-flume was analyzed, and the observed carbonate deposit arrangements were compared with expected water height in the flume, based on hydraulic calculations (Fig. 4). Flow is supercritical throughout the flume in all simulated situations (Figs. 4, 5; Supplementary Table S1).

**Table 1:** Parameters used to calculate the functioning of the Barbegal mill wheels and elbow flume.

Parameter	Description	Value
g	Gravity acceleration	9.81 m/s^2^
**Parameters for the basins**		
L_up_	Internal width of a millwheel basin	1.10 ± 0.05 m
H_up_	Water depth in the basins	variable [m]
Q_up_	Discharge (flow rate) through a basin	variable [m^3^/s]
**Parameters for the elbow flume**		
H_f_	Water depth at the end of the flume	variable [m]
L	Internal width of the flume without deposits	0.30 ± 0.02 m
H_c_	Critical depth for the flume	calculated from Q_f_ and L (Eq. 1) [m]
Ks	Strickler roughness coefficient of the flume	70–90 [m^1/3^/s]
V_inlet_	Inlet velocity of water into the flume	based on H_c_ and Q_f_ (critical flow) [m/s]
V_f_	Flume outlet velocity	variable [m/s]
Q_f_	Discharge (flow rate) in the flume	variable [m^3^/s]
**Parameters for the wheel**		
D	Diameter of the wheel	2.0 m
B	Outside width of the water wheel	0.85 m
b	Effective internal width of the buckets	0.75 m
N_c_	Critical velocity of the wheel rim	29.9 rpm (D = 2 m)
N_w_	Velocity of wheel rim when in operation	variable [rpm]
V_w_	Peripheric working velocity of the wheel	variable [m/s]
V_a_	Velocity of water striking the wheel	variable [m/s]
n	Number of buckets per wheel	25 (D = 2.0)
s	Wheel bucket surface area in cross-section	0.045–0.05 m^2^
Q_w_	Discharge delivered on the wheel	variable [m^3^/s]
T_c_	Ratio of N_w_/N_c_ for the wheel	0.2–0.4
T_v_	Ratio of V_a_/V_w_ for the wheel	2.0–2.5
T_b_	Mean filling ratio of buckets	0.3–0.4

Model values and their range have been estimated for wooden waterwheels from Roman time with a shape like those of Barbegal.

### Possible undershot mill and bypass

In a first model, the elbow-flume fed an undershot mill, installed with a steep slope creating a high-velocity feeding gutter to the mill wheel (Fig. 4a). This arrangement is unlikely, since overhang deposits would not form along the steep, long section of the flume (Figs. 3d, 4a). Instead, the water could only have overflown the side boards at the start of the flume, while water depth would have rapidly decreased in the long section downstream; this is the opposite of what was observed. In a second model where the elbow-flume functions as a bypass, hydraulic calculations show that overflow deposits are not expected to form in the flume (Fig. 4b). Water depth would be very low throughout the flume.

### Possible overshot mill

The only arrangement that fits the carbonate crust along the elbow-flume is that of Fig. 4c, where it feeds an overshot mill. In an elbow-flume with a short sloping leg and a long horizontal or gently dipping leg, water depth will be shallow in the former section and increase in the latter (Fig. 4c) in accordance with the observed distribution of overhang deposits (Fig. 3). This also sets the estimated length of the flume. The position of the axle window in the wheel pits indicates that mill wheels were in the center of the 4.9 m long pits (Fig. 5a)^13^. The length of the flume therefore cannot have exceeded 2.4 m and, considering the length of the parabola of falling water, was most likely 2.0–2.2 m (Fig. 5a). A 0.3–0.5 m long segment is therefore missing between segments E and R (Fig. 3e).

At first sight, it may seem unnecessary and even disadvantageous to use an elbow-flume in overshot mills. If the downstream leg is horizontal, the elbow segment decreases elevation of the water outlet to the wheel by at least 0.2 m (Figs. 4c, 5a), with an associated loss in potential energy and the necessity to use a smaller wheel than for a straight flume. Calculations of flow for an inclined straight flume ending at the same point as an elbow-flume with horizontal outrun leg (Fig. 4c) show that both give similar *H*_*f*_ and *V*_*f*_ at the outlet. The question is therefore why the mills used an elbow-flume, rather than a straight flume that would have been easier to construct. The unique shape of the elbow-flume of Barbegal suggests that it was specially designed for these mills. Hydraulic model calculations were carried out for an elbow-flume 2.1 m long as shown in Fig. 5a (Table 1; Supplementary Table S2). Compared to a straight flume, the elbow flume may ensure more stable critical flow conditions at the entrance, and may be less sensitive to transient operating conditions, as discussed hereafter.

### Hydraulic calculations for the flume in an overshot mill

Since the inclination angle of the elbow-flume outrun leg is unknown, the outlet flow velocities *V*_*f*_ and water depth *H*_*f*_ were modelled at variable inclination angles for a range of discharge values *Q*_*f*_. *Q*_*f*_ defines the critical depth *H*_*c*_ at the entrance of the flume and the associated headwater depth *H*_*up*_ in the feeding wheel pit basin (Fig. 5a). Both *H*_*c*_ and *H*_*up*_ increase with increasing discharge (Supplementary Table S4). *H*_*c*_ is defined by:

1 $${\text{H}}_{{\text{c}}} = \sqrt[3]{{\frac{{Q_{f}^{2} }}{{gL^{2} }}}}$$

*H*_*up*_ relates to *H*_*c*_, the critical water depth at the entrance of the flumes, by the subcritical solution of Eq. (2), describing specific energy conservation:

2 $$\frac{{Q_{up}^{2} }}{{2gL_{up}^{2} H_{up}^{2} }} + H_{up} = \frac{3}{2}H_{c}$$

where *Q*_*up*_ = *Q*_*f*_ and L_up_ is the width of the headwater basin. For wheels with a diameter of 2.0 m, *H*_*up*_ should not exceed a limiting depth of 0.25–0.35 m to avoid impairing rotation of the overhanging mill wheel (Fig. 5a)^43^. *Q*_*f*_ should be high enough to produce the observed high and overflowing water depths H_f_ in the runout leg of the flume, but low enough to keep *H*_*up*_ below this limiting depth. This limits discharge in the flume to *Q*_*f*_ < 0.12 m^3^/s. Examples of model results are shown in Fig. 4c (for *Q*_*f*_ = 0.1 m^3^/s), and calculated *V*_*f*_ and *H*_*f*_ for variable *Q*_*f*_ are plotted in Fig. 5b. The Strickler coefficient of roughness for a flume with carbonate deposits, needed for hydraulic calculations, was estimated as 70 < *Ks* < 90 m^1/3^ s^-1^. For an inclination angle of the flume outrun leg between -4° and 2° realistic values of *Q*_*f*_ between 0.05 and 0.11 m^3^/s and obtained *V*_*f*_ between 1.3 and 3 m/s were tested as possible operating conditions (Fig. 5b, Supplementary Table S2).

A realistic model should show overflowing conditions of the flume as indicated by the carbonate deposits. Side walls of the flume are 0.175 ± 0.003 m high, but regular overflow could be expected when *H*_*f*_ exceeds 0.16 m due to fluctuations of the water surface. Flume stratigraphy suggests a water depth *H*_*f*_ < 0.16 m when the flumes were relatively new but regular overflow (*H*_*f*_ > 0.16 m) at later stages after some carbonate had formed (Fig. 3). The values of *Q*_*f*_ and *V*_*f*_ for *H*_*f*_ exceeding 0.16 m are indicated in Fig. 5b for a new (*L* = 0.3) and a carbonate-incrusted flume (*L* = 0.26). In all models, flow remains supercritical throughout the flume (Figs. 4, 5; Supplementary Table S1) and overflow as shown by the deposits is therefore only attained at high discharge for a horizontal or counter-sloping flume (Figs. 4c, 5b). Such a counter-sloping flume, with the runout leg ending higher than the elbow, is possible if the discharge is high enough (*Q*_*f*_ > 0.055 m^3^/s for a − 4° slope). It will increase water depth in the flume, but at the same time decrease the outflow velocity, in combination limiting the permissive angle of the counter slope: a slightly counter-sloping flume (between − 1° and − 4°) at a relatively high discharge of 0.055 < *Q*_*f*_ < 0.11 m^3^/s is most likely (Fig. 5b). Flow can become subcritical through a hydraulic jump for higher counter-slope values, but in that case most of the flow from the flume is wasted by overflow, because of the height of the hydraulic jump. Therefore, this is not considered as a realistic option.

### Hydraulic calculations for an overshot mill wheel

In order to test the viability of our results further, the hydraulic properties of an overshot mill wheel that would fit in a 2.6 m deep wheel pit with an overshot elbow-flume was modelled. The necessary discharge delivered on the wheel *Q*_*w*_ and inflow velocity *V*_*a*_ striking the wheel can be estimated from *Q*_*f*_ and *V*_*f*_ calculated for the flume. *V*_*a*_ was derived from *V*_*f*_ and the slope of the flume outrun leg for an outflow water jet that was free-falling 0.2 m from the flume tip into the wheel buckets (Fig. 5b, Supplementary Table S2). *Q*_*w*_ is smaller than the flume discharge *Q*_*f*_ because of spillage. In Fig. 5b, an effect of spillage of 10% was used.

The discharge *Q*_*w*_ for a millwheel of 2 m diameter was modelled with 25 buckets, an internal bucket width *b* = 0.75 m and a bucket surface area in cross section of 0.045 < *s* < 0.05 m^2^, which would fit the observed carbonate fragments and the size of the preserved mill basins (Fig. 5a). According to Eqs. (3–7), wheel dimensions and parameters *T*_*c*_*, T*_*v*_ and *T*_*b*_ will delimit a range of values of *Q*_*w*_ and *V*_*a*_ for the wheel. *T*_*c*_ describes the ratio of N_*w*_ to the critical upper wheel velocity N_*c*_ (Eq. 4); *T*_*v*_ the ratio of the velocity of water striking the wheel *V*_*a*_ to *V*_w_ (Eq. 1); and *T*_*b*_ the filling ratio of wheel buckets. Since *T*_*c*_*, T*_*v*_ and *T*_*b*_ are unknown for Roman mills, values were estimated based on parameters for high-efficiency operation of late nineteenth to early twentieth century and modern overshot mills^32–34,44–46^ but adapted for a wooden wheel of the dimensions envisaged for Barbegal, as explained in the methods. This leads to a range of *Q*_*w*_ and *V*_*a*_ values indicated by the polygon in Fig. 5c (Supplementary Table S3 and Figure S2). Figure 5d compares the ranges of *Q*_*w*_ and *V*_*a*_ estimated for the wheel (Fig. 5c) to values calculated from an overflowing elbow flume (Fig. 5b). The most likely operating conditions are 0.5 < *Q*_*w*_ < 0.75 m^3^/s and 2.4 < *V*_*a*_ < 2.8 m/s for a wheel that was relatively wide (high *b* and *B*) with relatively full buckets (high *T*_*b*_) and relatively fast rotation (high *T*_*c*_; Fig. 5d). The results of hydraulic calculations for the flume are therefore confirmed by calculations for the mill wheel.

### Operation of the mill complex

In order to understand the reasons for the use of the elbow-flume in Barbegal, it is necessary to consider the operation of the entire mill complex. Operation of these mills faced unusual challenges, because of the limited water supply delivered by the aqueduct of 0.13 m^3^/s per mill train^42^ and because the shape and slope of the hill determined the maximum size of the mill wheel pits and the waterwheels, while the headwater basin for each mill also served as a tailwater basin, receiving the water from the wheel above it. The wheel size restricted the possible maximum water depth *H*_*up*_ in each tailwater basin to 0.25–0.35 m. Because of the slope of the hill, the basins had to be relatively small (4.9 ± 0.05 m long and 1.1 ± 0.05 m wide), carrying 1.22–1.94 m^3^ for a *H*_*up*_ of 0.24–0.34 m, corresponding to a discharge of 0.06 and 0.11 m^3^/s, respectively (Supplementary Table S4). For higher *Q*_*f*_ values, the water height in the headwater basin would increase to unrealistic values, with the risk of perturbing the rotation of the upstream wheel^43^, while the flume would overflow at its entrance. This setup means that there was a reduced head available to obtain the necessary discharge to operate the mills.

**Figure 6:**
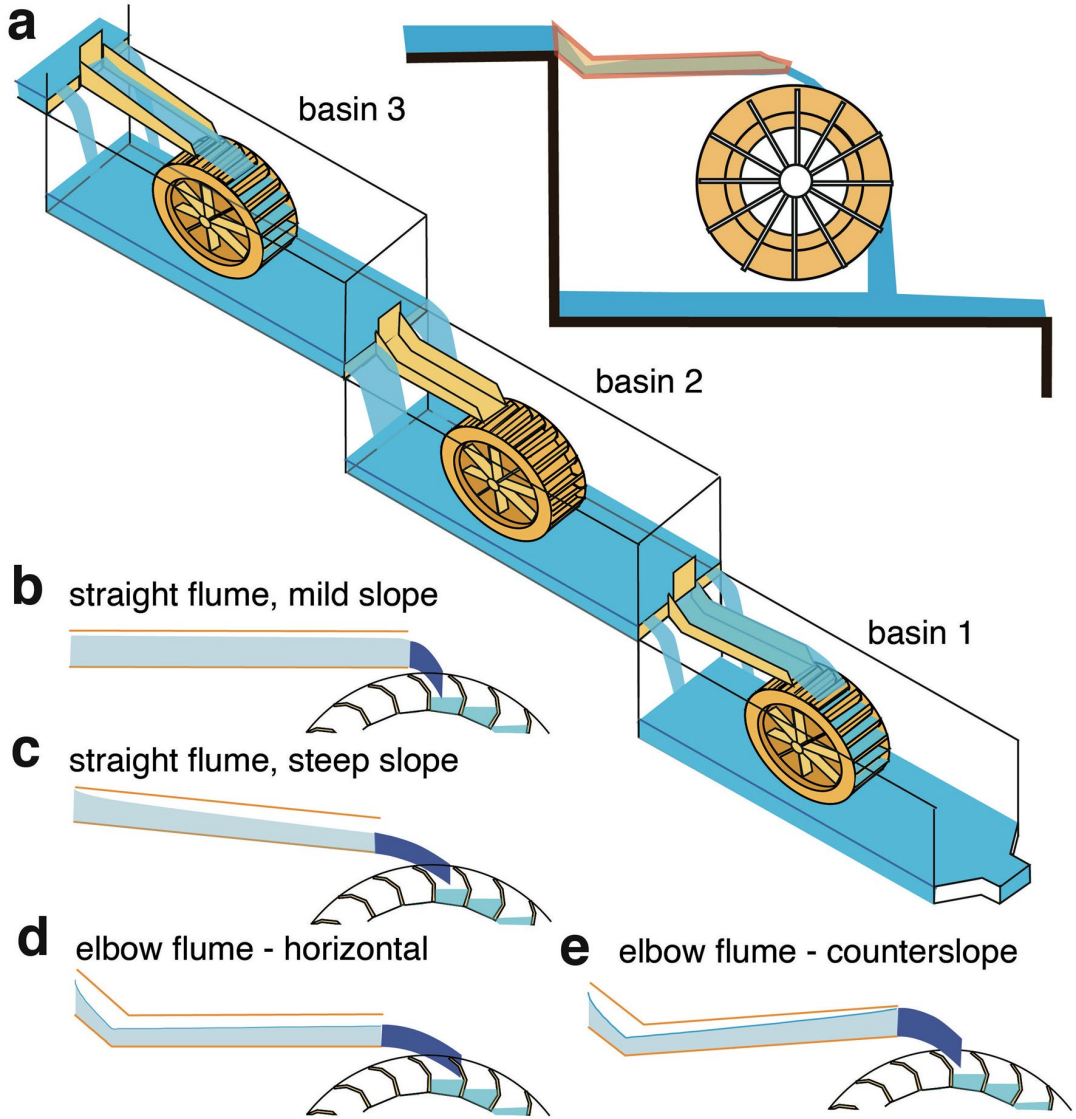
(**a**) Reconstruction of the three lowermost basins of the Barbegal complex, using elbow-flumes in basins 1 and 2. Overflow weirs beside the flumes allowed compensation for sudden fluctuations in discharge. (**b**)–(**e**) Summarized results of calculations on flow in four types of flumes, showing the presumed shape of outflow water jets.

Operation of the mills with wheels in series faces yet another consequence. Individual wheels had to be shut down and reactivated depending on demand. A full wheel could contain 7 full and 3 partially full buckets (Fig. 5a). Based on the wheel bucket dimensions *b*, *s* and *T*_*b*_ (Table 1) the millwheel could therefore contain 0.08–0.12 m^3^ of water when in operation. When a single wheel was shut down, this instantly increased the volume of water in the mill train downstream of it. Since each basin contained 1.22–1.94 m^3^ of water, this instantly elevated the water level in each subsequent basin by 4–10% (Fig. 6a). Similarly, the filling of an individual mill wheel would have led to a corresponding drop in headwater levels of downstream wheels along the train. Both effects could have caused a sudden change in discharge in the flumes, and possible damage to the mill mechanism, and this effect would be strongest for the lowermost flumes, since they have the largest number of upstream millwheels. The lowermost mills of the Barbegal complex therefore had to cope with a combination of low headwater levels and occasionally large fluctuations in discharge.

### The elbow-flume as a special design for the mill type

In modern mills, sudden changes in discharge in the flumes are avoided by the use of a large-volume headwater basin, and a wide overflow weir structure in the headwater dam, or by a weir in the feeding aqueduct limiting the discharge. This, however, was not possible in Barbegal. The basins were probably kept full to obtain a constant maximum head of water, and the downstream edge of the basins may have acted as a sharp-crested weir to drain excess water influx, and to buffer sudden fluctuations in water level (Fig. 6a). The length of this weir could have been optimized by keeping the flumes narrow, resulting in higher H_f_. Since the wheels were certainly wider that the flumes, this imposed the need to design a special flume type to obtain a maximum discharge from the relatively shallow headwater basins and to buffer sudden changes in discharge (Fig. 6b–e). For a similar discharge a straight flume, horizontal or with a mild slope (Fig. 6b) would have had the disadvantage of possible critical flow conditions at the end of the flume, with a possible instable regime due to the fact that the critical section would not be fixed by the geometry; it would result in higher H_up_ than a steeper flume with critical flow conditions at its entrance (Fig. 6c–e). A straight flume with steep slope (Fig. 6c) would provide critical flow conditions at the flume entrance and relatively low H_up_. However, the enhanced outlet velocity V_f_ could have produced an unsuitable outflow jet angle to fill the buckets efficiently, with enhanced spillage of water (Fig. 6c). An elbow-flume with steep upper leg would have ensured better and more stable critical control at the entrance of the flume, with water depth rapidly decreasing in the upper leg where the flow is accelerating and a regular and stable flow in the outrun leg of the flume (Fig. 6d). However, if the flume was horizontal, V_f_ and outflow jet angle would be similar to that of a steep straight flume (Fig. 6c,d). A counter-sloping elbow flume (Fig. 6e) would produce a reduced V_f_ at the end of the flume, providing a more suitably angled jet to fill the mill buckets, similar to that of a mildly sloping straight flume (Fig. 6b), but with the advantage of stable critical control at the flume entrance. An added advantage of a counter-sloping elbow-flume (Fig. 6e) is that in case of a sudden increase in discharge (due to halting of upstream mills) the counter-slope minimizes discharge variations in the wheel that could otherwise damage the mill machinery. The elbow flume design, with low boards in near-overflowing conditions would minimize the effects of flow surges while maximizing *Q*_*f*_ through an enhanced *V*_*inlet*_. The dip of the flume may even have been changeable if it was suspended from cables, providing the option to adapt *V*_*f*_. In this context it is important to note that the two lowermost basins of the Barbegal mills are 2.6 m deep while all other basins are 2.4 m deep. Possibly, the ~ 0.2 m high elbow-flumes were only used in these two lowermost basins, where the effect of upstream mill operation on discharge was largest, while straight flumes were used for the upstream mills (Fig. 6a). The difference in basin depth would imply that the lower basins were especially designed for the elbow flumes, which were therefore an integral part of the design of the entire mill complex. The use of elbow flumes also gives the possibility to install different water wheel diameters depending on basin depth and the type of installed millstones. Possibly, mill wheels were therefore of different size in different parts of the complex.

Summarizing, the elbow-flume seems to be an explicit design to solve technical problems specific to the lowest wheel pits of the Barbegal mills. It was a solution to counter the setbacks of the small volume of the headwater basins and potentially large variations in discharge, ensuring smooth operation of the mills.

## Discussion and perspectives

Ancient water technology is an important tangible aspect of cultural heritage throughout the world and Graeco-Roman water structures belong to the greatest technical achievements in antiquity^3,9^. Their remains are increasingly under threat of destruction by modern building activities and erosion and need to be studied before they are lost. This is not only of academic importance to improve our understanding of ancient technology. Water mills are environmentally friendly, cost-effective and highly efficient energy converters to exploit low-head hydropower, which presently remains mostly unused^32,33,45^. With an efficiency of up to 85%^32,33^, modern watermills can contribute significantly to the transfer from fossil fuels to renewable energy and can help to provide decentralized power and electricity at remote locations, with great potential in the developing world^46^. It is conceivable that simple, elegant solutions for complex technical problems, developed in antiquity and waiting to be discovered, may be applicable to modern and future water systems for sustainability. The unexpected discovery of the elbow-shaped flume in the mills of Barbegal illustrates this case in point.

The elbow-flume seems to be unique in the history of hydraulics: no comparable flumes are known from ancient or modern mills. It was probably designed to meet two specific purposes: (1) to deliver a maximum discharge to a wheel with the correct outlet velocity and angle through a necessarily narrow flume when only a limited headwater elevation was available, and (2) to dampen fluctuations in water level caused by intermittent operation of upstream mills. As such, the elbow-flume of the Barbegal mills is a unique witness of the prowess and advanced technological stage of Roman hydraulic engineering.

Carbonate deposits in ancient water systems serve another purpose. Many aquifers in the Mediterranean are currently being depleted and/or are polluted posing serious water problems^47,48^. The study of carbonate deposits in ancient water systems can provide long-term information on the discharge and water composition of crucial springs^20^. This knowledge can be useful for hydrologists to identify which springs can be regenerated or reused, or how depletion can be mitigated, for instance as a result of climate change^49^. This study therefore shows that the study of freshwater carbonates in ancient water systems can make an important contribution, not only to the history of science but also to provide data on the local palaeoenvironment.

## Methods

### Macroscopic characterization

The mill basins or wheel pits are 1.1 m wide and 4.9 m long (Fig. 1). The upper mill basins are 2.4 m deep, and the two lowest ones 2.6 m—all measures with an estimated accuracy of ± 0.05 m. 142 carbonate fragments were labelled N1-N142^30^. The sides or cross-sections of these fragments, where the internal stratigraphy is visible, are labelled f(-) (Fig. 3, Supplementary Fig. S1). The fragments were studied, and some sampled, in the Museum of Antiquities in Arles where they are curated. Millrun flumes had an inner diameter of 0.3 ± 0.02 m, determined from the width of preserved carbonate segments from flume bottoms (Fig. 2a). The elbow-flume modelled here had sidewalls 0.175 ± 0.003 m high determined from three segments (Fig. 3).

### Microscopic characterization

Thin sections of the carbonate deposits were microscopically investigated by standard methods as described before^15,30^.

### Hydraulic modelling

Two interlinked types of hydraulic modelling were performed, flow in millrun flumes (1) and through a mill wheel (2). Parameters are defined in Table 1.

### Flumes

Hydraulic calculations for flumes served to estimate water depth (*H*_*f*_) and outflow velocity (*V*_*f*_) for a given discharge. The model flume was 0.3 m or 0.26 m wide and 2.1 m long, with an estimated roughness coefficient of 70 < *Ks* < 90 m^1/3^ s^−1^ for the sides and bottom. The lower value corresponds to a roughness height on flume walls and bottom of about 3 mm, representing irregular rough deposits. The higher value corresponds to a smoother wall surface with roughness height of about 0.5 mm, such as on new surfaces before deposits. During the lifetime of operation of a flume, roughness may have varied from smooth to rough. Both elbow-shaped and straight flumes were modelled. The angle between the upper leg and the outrun leg was 40° ± 1° for elbow-flumes. Due to the rectangular shape of the flume cross section, hydraulic calculations were straightforward, based on classical equations for one-dimensional steady free surface flows^50,51^. All hydraulic calculations were performed using an iterative procedure with an Excel spreadsheet. It was assumed that water was freely flowing from the upper wheel pit basin to the flume, with a critical section at the entrance of the flume. Calculations were carried out from upstream to downstream for supercritical flow in the flume. In case of a transition towards subcritical flow through a hydraulic jump, the subcritical part of the flow is calculated from the downstream condition (critical section at the exit) to the upstream, until hydraulic jump relationships are encountered. All calculations were made for a flume with an open end and similar width as the upstream section, as the final part of a possible nozzle was not preserved. Carbonate deposits formed inside the flume would gradually reduce the internal width *L* from 0.30 m (unused) to 0.26 m after seven years of use.

### Millwheels

The dimensions of the preserved mill basins and carbonate fragments were used to determine the most likely geometry of the mill wheels. The reconstructed wheel geometry was then used to calculate the possible discharge *Q*_*w*_ of water passing through the wheels, and the necessary velocity *V*_*a*_ of water striking the wheels. By combining the calculations of the wheels and the millrun flumes, the operation conditions of the mill wheels were determined. All parameters are summarized in Table 1. The maximum external width of the wheels was estimated as B = 0.85 m, since sufficient space must be left between the wheel and the walls of the 1.1 m wide basins. Assuming the mills are overshot and leaving sufficient space between the wheels and the tip of the flumes and between the wheels and the tailwater, the maximum diameter D of a wheel is 2.0 m in a 2.6 m deep pit, and 1.8 m in a 2.4 m deep pit. Carbonate fragments, presumably from the wheel and with the same stratigraphy as the flumes (Fig. 2c,d) suggest that the wheels were supplied with curved buckets^30^, an arrangement also seen in some other Roman mill wheels^35^.

For any given diameter *D*, a waterwheel with curved buckets has a critical rotation velocity (*N*_*c*_), above which water loss and danger to the structure by centrifugal forces becomes problematic. *N*_*c*_ is calculated as^44^:

3 $$N_{c} = 42.3/\sqrt D$$

For *D* = 2 m, *N*_*c*_ = 29.9 rpm. The operating rotation velocity of mills (*N*_*w*_) is related to the critical velocity *N*_*c*_ by

4 $${\text{T}}_{{\text{c}}} = {\text{N}}_{{\text{w}}} /{\text{N}}_{{\text{c}}}$$

Using values for most efficient wheel velocity^32,34,44,46^, *T*_*c*_ should be in the range of 0.2–0.47. For a Roman wheel as that of Barbegal, *N*_*w*_ cannot reach very high values since Roman wheels lacked the balancing and bearings of modern mill wheels^34^. We therefore assume a range of 0.2 < *T*_*c*_ < 0.4. Using descriptions of other Roman wheels, notably that of Hagendorn^35^ and the dimensions of segments found at Barbegal (Fig. 2c), the number of buckets per wheel n was estimated as 25 for *D* = 2 m. The discharge *Q*_*w*_ flowing through the wheel was then estimated as

5 $${\text{Q}}_{{\text{w}}} = {\text{ N}}_{{\text{w}}} {\text{b s T}}_{{\text{b}}} {\text{n}}/60$$

*T*_*b*_, the filling ratio of wheel buckets is generally between 0.3 and 0.6^32,34,44,46^ but cannot exceed 0.4 for the Barbegal wheel because of the inferred shape of the wooden buckets (0.3 < *T*_*b*_ < 0.4).

In order for a waterwheel to operate under optimal conditions, the inflow speed *V*_*a*_ of the water must exceed the working velocity *V*_*w*_ of the wheel, expressed as the ratio *T*_*v*_

6 $${\text{T}}_{{\text{v}}} = {\text{V}}_{{\text{a}}} /{\text{V}}_{{\text{w}}}$$

7 $${\text{with}}\;{\text{V}}_{{\text{w}}} = {\text{ N}}_{{\text{w}}} \pi {\text{D}}/60$$

*T*_*v*_ is estimated to be in the range of 1.25–2.5 for modern mills^32–34,44–46^. For the Barbegal wheel, a relatively high range is likely, estimated as 2.0 < *T*_*V*_ < 2.5

Summarizing, discharge Q_w_ and inflow speed V_a_ can be calculated from just five parameters: *D, b, T*_*c*_*, T*_*v*_ and *T*_*b*_ using Eqs. (3–7).

### Stable isotopes

Analyses of stable oxygen and carbon isotope were carried out at the University of Innsbruck. Polished slabs of all samples were micromilled at 0.2 mm intervals in traces 5 mm wide and parallel to the lamination. The sample powders were analysed using a semi-automated device (Gasbench II) linked to a ThermoFisher Delta V Plus isotope ratio mass spectrometer. Isotope values, expressed as δ^18^O and δ^13^C, are reported on the VPDB scale and long-term precision is better than 0.1‰ for both δ^13^C and δ^18^O. Further details are given in^15,16^.

## Supplementary information

Supplementary Information.
