# Supplementary material for: Reconstructing the hydraulics of the world’s first industrial complex, the second century CE Barbegal watermills, France
Source: Sci Rep. 2020 Oct 21;10:17917. doi: 10.1038/s41598-020-74900-5 (PMC7578838; doi:10.1038/s41598-020-74900-5)
Supplement: Supplementary file 1 — Supplementary Information. [file 41598_2020_74900_MOESM1_ESM.pdf]

Supplementary Information

Reconstructing the hydraulics of the world's first industrial complex, the second century CE Barbegal watermills, France

Cees W. Passchier[1], Marcel Bourgeois[2], Pierre-Louis Viollet[3], Gül Sürmelihindi[1], Vincent Bernard[4], Philippe Leveau[5], Christoph Spötl[6]

[1] Department of Earth Sciences, Johannes Gutenberg University, 55128 Mainz, Germany

[2] Vicat Group, 38081 L'Isle d'Abeau Cedex, France

[3] Société Hydrotechnique de France, 25 rue des Favorites, 75015 Paris, France

[4] Université Rennes 1, CNRS, UMR 6566 CReAAH, Rennes, France

[5] Aix Marseille Université, CNRS, CCJ, UMR 7299, 13094, Aix-en-Provence, France

[6] Institute of Geology, University of Innsbruck, Innrain 52, 6020 Innsbruck, Austria

Figure S1. Internal stratigraphy of the elbow flume

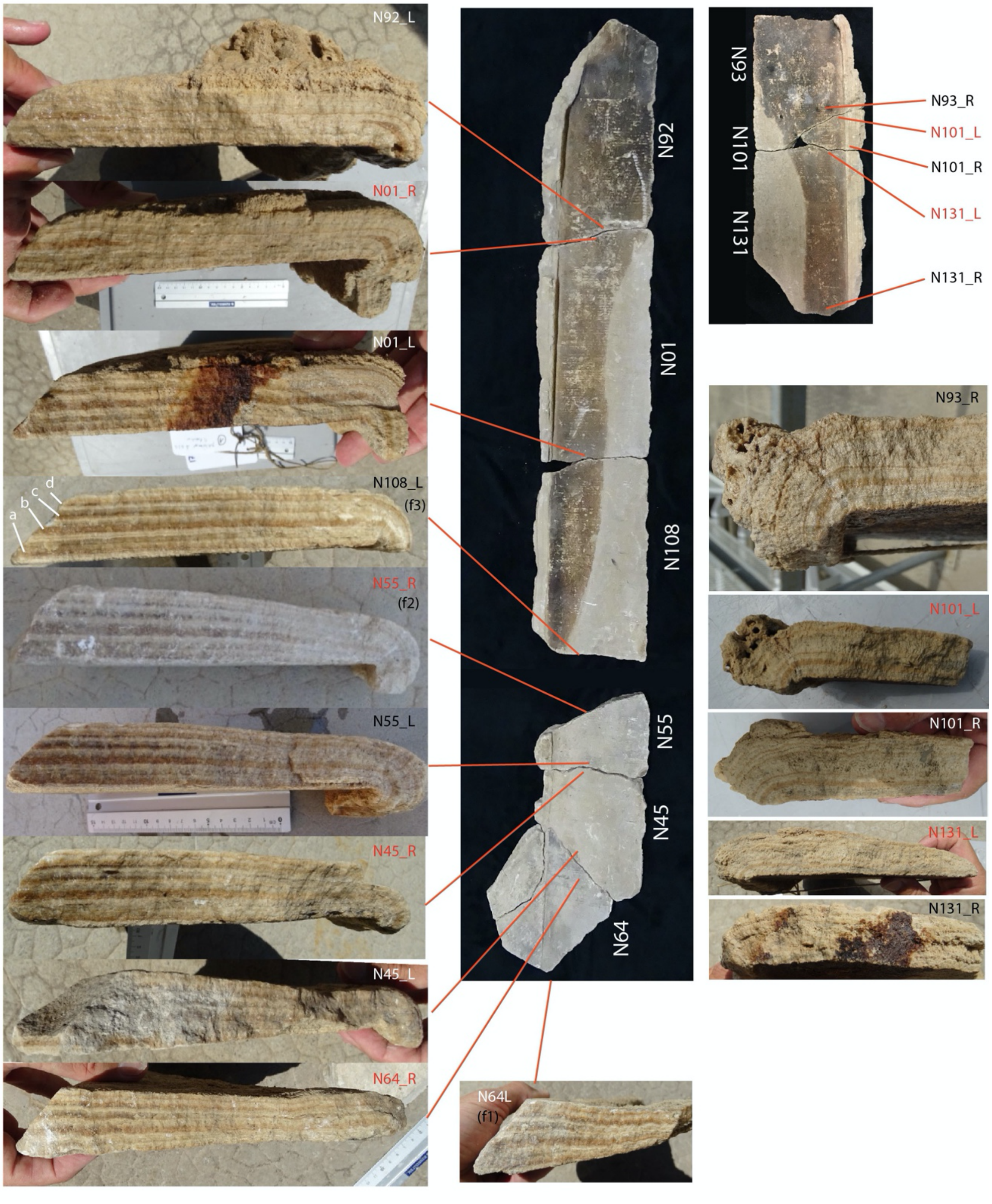

Fig. S1. Internal stratigraphy of segments R, E and L. Red numbers refer to images that have been mirrored in order to show the same geometry as the non-inverted ones. A gradual change in the profile of the carbonate deposits is visible, from a low, triangular shape indicating low water levels in the top of the elbow (upstream) to overflowing, indicated by the uniform thickness of the carbonate crust downstream. Note late porous carbonate deposits with plant remains in sections N92L, N126L, and R, N93R and N101L and R. Black labels f1, f2 and f3 refer to sections discussed in the text and in Fig. 3.

Figure S2. Plot of the value range of Q_w and V_a based on parameters of the mill wheel

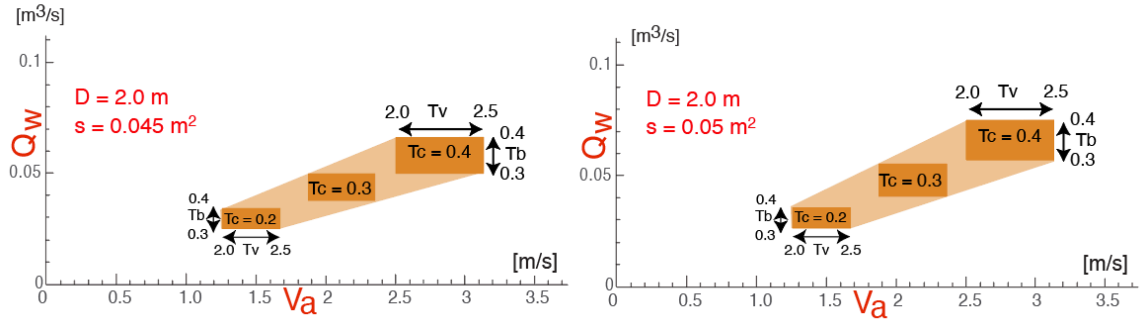

Figure S2. Plot of the range of Q_w and V_a for a millwheel of Barbegal based on parameters D, b, T_v, T_a and T_c, as explained in the method sections. This figure plots the values presented in Table S3. Each polygon is based on ranges of T_c, T_v and T_b as indicated. A combined polygon for values of $0.045<s<0.05$ was used to construct Figure 5c in the main text.

Table S1. Results of calculations for the elbow flume for Figure 4

		Angle and slope of elbow-flume								
**Q_f [m^3/s]**	H_f [m], V_f [m/s]	0.052 (3˚)	0.035 (2˚)	0.017 (1˚)	0 (0˚)	-0.02 (-1˚)	-0.04 (-2˚)	-0.05 (-3˚)	-0.07 (-4˚)	**H_c [m]**
0.03	H_f	0.047	0.049	0.052	0.056	0.061	0.068	0.081	-	0.101
	V_f	2.149	2.039	1.920	1.789	1.640	1.466	1.235	-	
0.04	H_f	0.057	0.060	0.063	0.067	0.073	0.080	0.092	0.137	0.122
	V_f	2.342	2.229	2.108	1.976	1.828	1.659	1.450	0.974	
0.05	H_f	0.067	0.070	0.074	0.079	0.085	0.093	0.104	0.129	0.141
	V_f	2.483	2.369	2.247	2.114	1.967	1.800	1.598	1.295	
0.06	H_f	0.077	0.081	0.085	0.090	0.096	0.105	0.117	0.139	0.160
	V_f	2.595	2.480	2.356	2.223	2.076	1.910	1.713	1.436	
0.07	H_f	0.087	0.091	0.095	0.101	0.108	0.117	0.129	0.151	0.177
	V_f	2.687	2.571	2.447	2.313	2.166	2.001	1.807	1.542	
0.08	H_f	0.096	0.101	0.106	0.112	0.119	0.128	0.141	0.164	0.194
	V_f	2.765	2.649	2.524	2.390	2.244	2.079	1.886	1.628	
0.09	H_f	0.106	0.110	0.116	0.122	0.130	0.140	0.153	0.176	0.209
	V_f	2.834	2.716	2.592	2.458	2.311	2.147	1.955	1.701	
0.1	H_f	0.115	0.120	0.126	0.132	0.141	0.151	0.165	0.189	0.225
	V_f	2.894	2.777	2.652	2.518	2.371	2.208	2.016	1.765	
0.11	H_f	0.124	0.129	0.135	0.143	0.151	0.162	0.177	0.201	0.239
	V_f	2.949	2.832	2.707	2.572	2.426	2.263	2.072	1.823	
0.12	H_f	0.133	0.139	0.145	0.153	0.162	0.173	0.188	0.213	0.254
	V_f	2.999	2.881	2.756	2.622	2.476	2.313	2.122	1.874	

Table S1. Results of calculations used for flumes in Fig. 4c in the main text. All values are based on a 2.1 m long elbow gutter with L=0.3 m and Ks = 70 $m^{1/3}s^{-1}$

Table S2. Results of calculations for the elbow flume for Figure 5b

		L=0.3		L=0.3		L=0.26		L=0.26		L=0.3	L=0.3
		Ks=70		Ks=90		Ks=70		Ks=90		Ks=70	KS=90
Q_f	angle	V_f [m/s]	H_f [m]	V_f [m/s]	H_f [m]	V_f [m/s]	H_f [m]	V_f [m/s]	H_f [m]	V_a [m]	V_a [m]
0.11	-4	1.82	0.201	2.07	0.178	1.83	0.231	2.11	0.201	2.68	2.86
0.1	-4	1.77	0.189	2.01	0.166	1.77	0.217	2.06	0.187	2.66	2.82
0.09	-4	1.70	0.176	1.96	0.153	1.71	0.202	2.00	0.173	2.61	2.79
0.08	-4	1.63	0.164	1.89	0.141	1.64	0.187	1.94	0.159	2.57	2.74
0.07	-4	1.54	0.151	1.82	0.128	1.56	0.173	1.87	0.144	2.51	2.69
0.06	-4	1.44	0.139	1.74	0.115	1.46	0.158	1.78	0.129	2.45	2.64
0.05	-4	1.29	0.129	1.64	0.102	1.32	0.146	1.69	0.144	2.36	2.57
0.11	-2	2.26	0.162	2.45	0.150	2.30	0.184	2.50	0.170	3.01	3.15
0.1	-2	2.21	0.151	2.40	0.139	2.24	0.171	2.44	0.157	2.97	3.11
0.09	-2	2.15	0.140	2.34	0.128	2.18	0.159	2.39	0.145	2.92	3.07
0.08	-2	2.08	0.128	2.28	0.117	2.12	0.145	2.33	0.132	2.87	3.02
0.07	-2	2.00	0.117	2.21	0.106	2.04	0.132	2.26	0.119	2.81	2.97
0.06	-2	1.91	0.105	2.13	0.094	1.95	0.118	2.18	0.106	2.75	2.91
0.05	-2	1.80	0.093	2.03	0.082	1.85	0.104	2.08	0.092	2.68	2.84
0.11	0	2.57	0.143	2.75	0.134	2.61	0.162	2.80	0.151	3.24	3.39
0.1	0	2.52	0.132	2.70	0.124	2.56	0.150	2.75	0.140	3.20	3.35
0.09	0	2.46	0.122	2.64	0.114	2.50	0.139	2.69	0.129	3.16	3.30
0.08	0	2.39	0.112	2.58	0.103	2.43	0.126	2.63	0.117	3.10	3.25
0.07	0	2.31	0.101	2.51	0.093	2.36	0.114	2.56	0.105	3.04	3.20
0.06	0	2.22	0.090	2.43	0.082	2.27	0.102	2.48	0.093	2.98	3.13
0.05	0	2.11	0.079	2.34	0.071	2.16	0.089	2.39	0.081	2.89	3.07
0.11	2	2.83	0.129	3.00	0.122	2.87	0.147	3.05	0.139	3.45	3.59
0.1	2	2.78	0.120	2.95	0.113	2.82	0.136	3.00	0.128	3.41	3.55
0.09	2	2.72	0.110	2.90	0.103	2.76	0.125	2.95	0.177	3.36	3.51
0.08	2	2.65	0.101	2.84	0.094	2.69	0.114	2.89	0.107	3.31	3.46
0.07	2	2.57	0.091	2.77	0.084	2.62	0.103	2.82	0.095	3.24	3.41
0.06	2	2.48	0.081	2.69	0.074	2.53	0.091	2.74	0.084	3.17	3.34
0.05	2	2.37	0.070	2.59	0.06	2.42	0.08	2.65	0.073	3.09	3.26

Table S2. Results of calculations of V_f and H_f used in Fig. 5b in the main text. All values are based on an elbow gutter 2.1m long for variable Q_f (0.5-1.1 m^3/s) and slope of the outrun leg of the flume (-4° to 2°). Values are given for two flume widths, L=0.3 and L=0.26, the latter for a flume that is partially filled with carbonate. For each value of L, two values of the Strickler coefficient Ks are considered (70 and 90). The last two columns are final velocity values (V_a) derived for V_f and the slope of the flume, for water falling 0.2 meter towards the wheel.

Table S3. Q_w and V_a calculated for the wheel for Fig. 5c

S = 0.045 m²

T_c	T_v	T_b	Q_w	V_a
0.2	2	0.3	0.025	1.25
0.2	2.5	0.3	0.025	1.57
0.2	2	0.4	0.034	1.25
0.2	2.5	0.4	0.034	1.57
0.3	2	0.3	0.038	1.88
0.3	2.5	0.3	0.038	2.35
0.3	2	0.4	0.050	1.88
0.3	2.5	0.4	0.050	2.35
0.4	2	0.3	0.050	2.50
0.4	2.5	0.3	0.050	3.13
0.4	2	0.4	0.067	2.50
0.4	2.5	0.4	0.067	3.13

S = 0.05 m²

T_c	T_v	T_b	Q_w [m³/s]	V_a [m/s]
0.2	2	0.3	0.028	1.25
0.2	2.5	0.3	0.028	1.57
0.2	2	0.4	0.037	1.25
0.2	2.5	0.4	0.037	1.57
0.3	2	0.3	0.042	1.88
0.3	2.5	0.3	0.042	2.35
0.3	2	0.4	0.056	1.88
0.3	2.5	0.4	0.056	2.35
0.4	2	0.3	0.056	2.50
0.4	2.5	0.3	0.056	3.13
0.4	2	0.4	0.075	2.50
0.4	2.5	0.4	0.075	3.13

Table S3. Values of Q_w and V_a calculated for a 2 m diameter mill wheel of b = 0.75m and N_c = 29.9 for two values of bucket surface area s, and a range of T_c, T_v and T_b. These values were used to constrict the polygon for mill wheel parameters in Fig. 5c of the main paper. Supplementary Figure S2 explains the procedure to plot the polygon in Figure 5c and 5d.

Table S4. Water heights H_c and H_{up} as a function of Q_{up}

$Q_{up} = Q_f$ (m^3/s)	Critical height H_c at the entrance of the flume (m)	Water height in the upstream basin H_{up} (m)
0.06	0.160	0.237
0.1	0.224	0.333
0.15	0.294	0.436

Table S4. Water height in the upstream basin for different values of the flow rate, based on equation 2 in the main text.
